# Creation and validation of a proteome-wide yeast library for protein detection and analysis

**DOI:** 10.1242/jcs.263848

**Published:** 2025-09-01

**Authors:** Din Baruch, Ioannis Tsirkas, Ehud Sass, Benjamin Dubreuil, Yeynit Asraf, Amir Aharoni, Maya Schuldiner, Ofir Klein

**Affiliations:** ^1^ Department of Molecular Genetics, Weizmann Institute of Science, Rehovot 7610001, Israel; ^2^ Department of Life Sciences and the National Institute for Biotechnology in the Negev, Ben-Gurion University of the Negev, Be'er Sheva, 84105, Israel

**Keywords:** *Saccharomyces cerevisiae*, Proteome-wide library, HA tag, SWAT approach, Single-chain fragment variable, scFv

## Abstract

A significant challenge in cell biology is to uncover the function of uncharacterized proteins. Surprisingly, a quarter of the proteome is still poorly understood even in the most well-studied model organisms. Systematic methodologies, including the use of tagged protein collections, have emerged as a powerful approach to address this gap. Despite the availability of proteome-wide collections featuring various fused proteins, the impact of different tags on protein function highlights the need for diversifying the tags used for functional genomic studies. To rise to this challenge, we created a proteome-wide collection of yeast strains in which proteins are N-terminally tagged with the broadly utilized and compact hemagglutinin (HA) epitope. We showcase the potential uses of our library for systematically evaluating protein size, abundance and localization using an *in vivo* labeling approach. Our characterization underscores the potential utility of a proteome-wide HA-tagged library in revealing novel aspects of cell biology, providing an additional powerful tool for functional genomics.

## Teaser

**Summary:** A new yeast library where each protein is N-terminally tagged with a compact HA epitope, enabling systematic *in vivo* and *in vitro* measurement of protein size, abundance, and localization.

## INTRODUCTION

In the post-genomic era, a paramount challenge still lies in mapping the functions of all uncharacterized proteins (Rocha et al., 2023). Despite the fact that both the yeast and human genomes were sequenced decades ago (Goffeau et al., 1996; International Human Genome Sequencing Consortium, 2004), there are still thousands of proteins, constituting up to a quarter of the proteome, with unknown or poorly understood functions (Cohen et al., 2022; Eisenhaber, 2012; Peña-Castillo and Hughes, 2007; Rocha et al., 2023; Wood et al., 2019). Uncovering the function of uncharacterized proteins can be approached either one protein at a time or through systematic, high-throughput strategies, collectively termed functional genomics. Among the various systematic methodologies, pulldown assays for interactor mapping, and localization studies are commonly employed. These methods often rely on the introduction of single common epitope(s) by fusion of each protein to genetically encoded tags.

An established tool allowing for the simultaneous study of thousands of proteins is the use of libraries – collections of strains in which each gene is genetically manipulated in a similar manner. In tagged libraries, each protein is genetically fused to a tag of interest, one protein at a time. Although systematic libraries have been created for several organisms (see, for example, Bischof et al., 2013; Cho et al., 2022) the most utilized eukaryotic model organism in library creation is the baker's yeast *Saccharomyces cerevisiae* (hereafter yeast), which is a popular and well-studied model organism for functional genomics due to the availability of extensive genetic and genomic tools, and given that approximately two-thirds of its proteome is conserved in humans (Cohen et al., 2022)*.*

Many yeast libraries have been created with different tags, including fluorescent proteins (FPs) (see, for example, Huh et al., 2003; Meurer et al., 2018; Weill et al., 2018) and affinity purification tags [such as the tandem affinity purification (TAP) tag; Ghaemmaghami et al., 2003]. The vast majority of the libraries rely on big tags (15–30 kDa), which raise a concern as to their effect on protein folding, complex assembly, stability, targeting and function (Beatty and López, 2023; Terpe, 2003; Zhao et al., 2013).

Several small and unstructured tags have been engineered. A particularly useful one is the hemagglutinin (HA) tag, which is just nine amino acids in length. The HA tag is not only effective, like other small tags, for immunoprecipitation (IP) studies, but was also recently used for *in vivo* visualization in combination with a newly developed genetically encoded single-chain fragment variable (scFv) fused to a FP (Murakawa et al., 2022; Tsirkas et al., 2022; Zhao et al., 2019). Recognizing these advantages of the HA tag, we created a proteome-wide yeast library where each protein is N-terminally (N′) tagged with HA. For library creation, we utilized the SWAp-Tag (SWAT) strategy, which revolutionized the speed and capacity to create new yeast libraries (Meurer et al., 2018; Weill et al., 2018; Yofe et al., 2016). We demonstrate the HA collection efficacy in functionally diverse applications including the analysis of protein size, abundance and localization, highlighting the beneficial effect of the HA tag in comparison to the green fluorescent protein (GFP) tag on native protein localization for a subgroup of proteins. Overall, our efforts provide the yeast community with a new and powerful tool for functional genomics, that will be freely distributed.

## RESULTS

### Creation and validation of an N′ HA-tagged library

Until now, the yeast proteome-wide collections that have been constructed for protein visualization (Huh et al., 2003; Meurer et al., 2018; Weill et al., 2018) or purification (Ghaemmaghami et al., 2003) have mostly relied on large tags such as GFP, mCherry, mScarlet-I, mNeonGreen and TAP. These tags, although effective, are large and often highly structured. Previously, we have created a yeast strain collection addressing this issue, where each protein is tagged at the C-terminus (C′) with a Myc–HRV–3xFlag cassette, summing up to a tag of 5.2 kDa (2.7 kDa post HRV cleavage) (Reinhard et al., 2024). This library holds the benefit of having each gene under its endogenous promoter, yet the C′ tag might interfere with C′ targeting sequences, post translational modifications on the C′ and it also disrupts the native terminator. Thus, we set out to complement this with the creation of a proteome-wide N′ HA-tagged yeast library. To further diversify the tools available for functional genomics, we chose to use the HA tag. Like the Myc and Flag tags, the HA is a small tag that has been widely used and is suitable for many applications (Brizzard, 2008; Zhao et al., 2013). Yet, the HA tag is less charged (−2, pH 7) than the Myc and flag tags (−3, pH 7 each) and thus reduces the charge effect of the tagged protein.

**Fig. 1.**
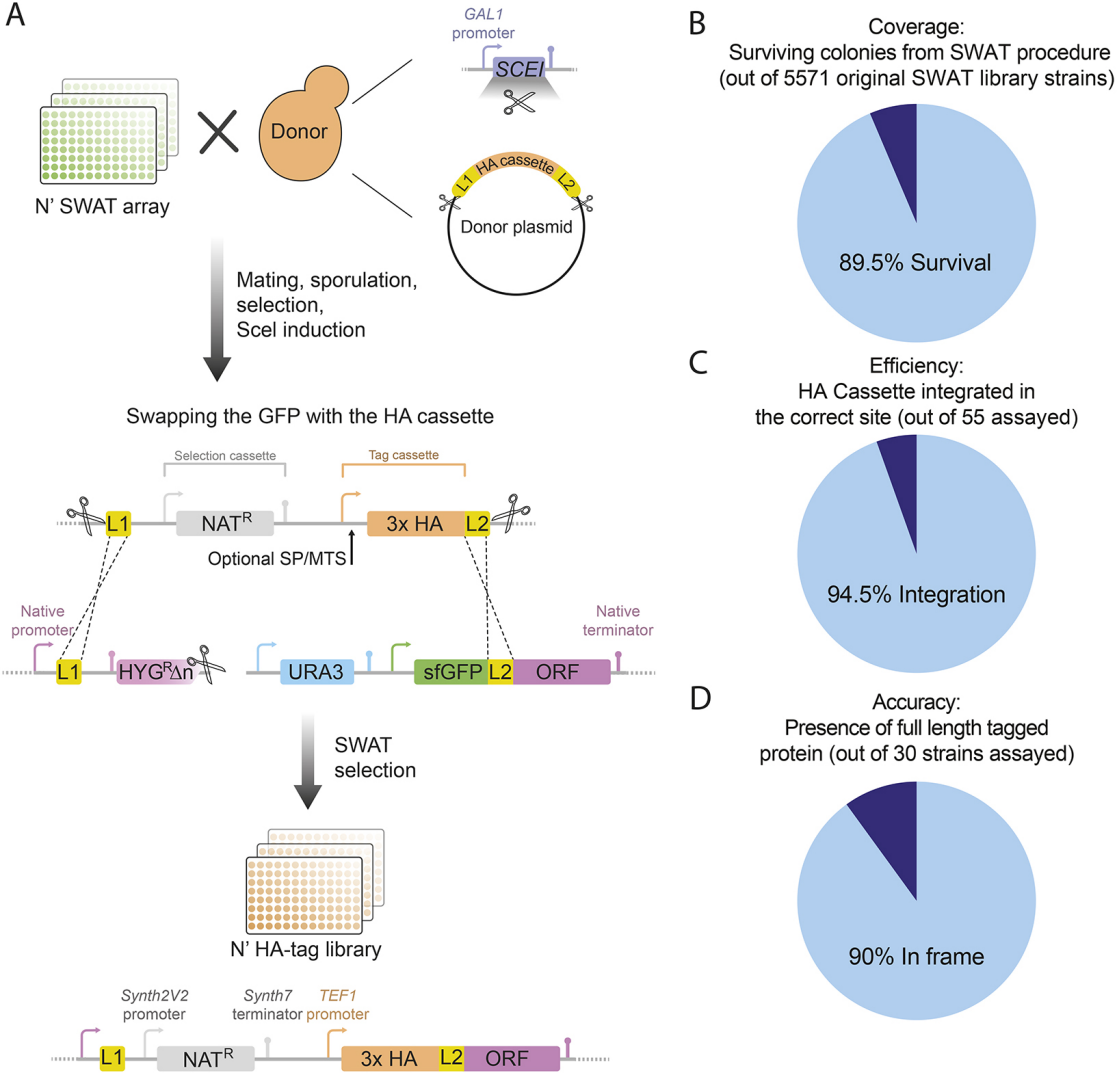
**Creation and validation of an HA tag yeast library.** (A) An illustration of the HA library creation. The acceptor N′-SWAT library (harboring the SWAT module, which includes the L1 and L2 linkers, Ura3 selection and a I-SceI restriction site) was crossed with a donor strain harboring a donor plasmid (encoding for the HA tag and a Nat selection, situated between the L1 and L2 linkers and flanked by I-SceI restriction sites) and a galactose inducible I-SceI restriction enzyme. An automated mating and selection procedure created an intermediate library carrying all traits mentioned above. Induction of I-SceI expression (by plating on galactose) resulted in double-strand breaks in both the donor plasmid and the genomic SWAT module, leading to homologous recombination between the L1 and L2 linkers, replacing the SWAT module with the HA cassette. Subsequent selection for Nat and against Ura3 resulted in the desired HA library. (B) Assessment of the efficiency of the SWAT procedure (coverage) was performed by measuring the number of colonies that passed all the automated mating, selection and SWAT procedures. A pie chart represents that 89.5% of yeast strains from the original library survived. (C) Assessment of the HA cassette SWAT efficiency was performed by check PCR upstream to randomly chosen genes. The pie chart demonstrates that 94.5% of genes had a successful integration (*n*=55). (D) Assessment of the efficiency of in-frame integration (accuracy) was performed by evaluating the molecular mass of tagged proteins. The pie chart demonstrates that, for randomly chosen soluble proteins analyzed by SDS-PAGE, 90% of proteins run at the expected molecular mass, and therefore we assume that they represent in-frame integration of the HA tag (*n*=30).

To create the library, we used the SWAT strategy, which employs an acceptor library with an interchangeable cassette that can be replaced with a tag of choice by homologous recombination to rapidly and efficiently generate new yeast libraries (Yofe et al., 2016) (Fig. 1A). The resulting library comprises a systematic collection of proteins N′ fused to a 3xHA epitope tag, positioned under the control of the strong and constitutive *TEF1* promoter and a nourseothricin (Nat) resistance cassette, in *MATa* mating type, ensuring compatibility for downstream applications (Fig. 1A).

In line with what as seen with generation of previous SWAT-based generated libraries (Weill et al., 2018), this process had a very high efficiency, with 89.5% of yeast strains from the original library surviving the procedure (Fig. 1B; Table S1). Moreover, 94.5% of the 55 sampled strains exhibited successful integration of the HA tag, as confirmed by PCR analysis (Fig. 1C; Tables S2, S7). The accuracy of in-frame integration was further assessed by SDS-PAGE, revealing that 90% of the 30 randomly selected soluble proteins were detected at the expected molecular mass of the fusion protein (Fig. 1D; Fig. S1). Furthermore, all 36 colonies tested for the loss of GFP signal from the original acceptor cassette, had indeed lost it. Altogether, we approximate that out of 5571 yeast strains present in the original SWAT library, a minimum of 4486 strains are bona fide HA-tagged proteins in our collection. This high coverage underscores the effectiveness of the SWAT procedure as a method to create novel libraries. More importantly, it makes our N′ HA-tagged library a powerful tool for systematic proteome-wide functional genomic studies. This library can provide a reliable resource for downstream systematic applications, such as tracking protein size, abundance and localization, enabling a comprehensive analysis across various experimental conditions.

### Rapid examination of post-translational modifications using the N′ HA-tagged strains

**Fig. 2.**
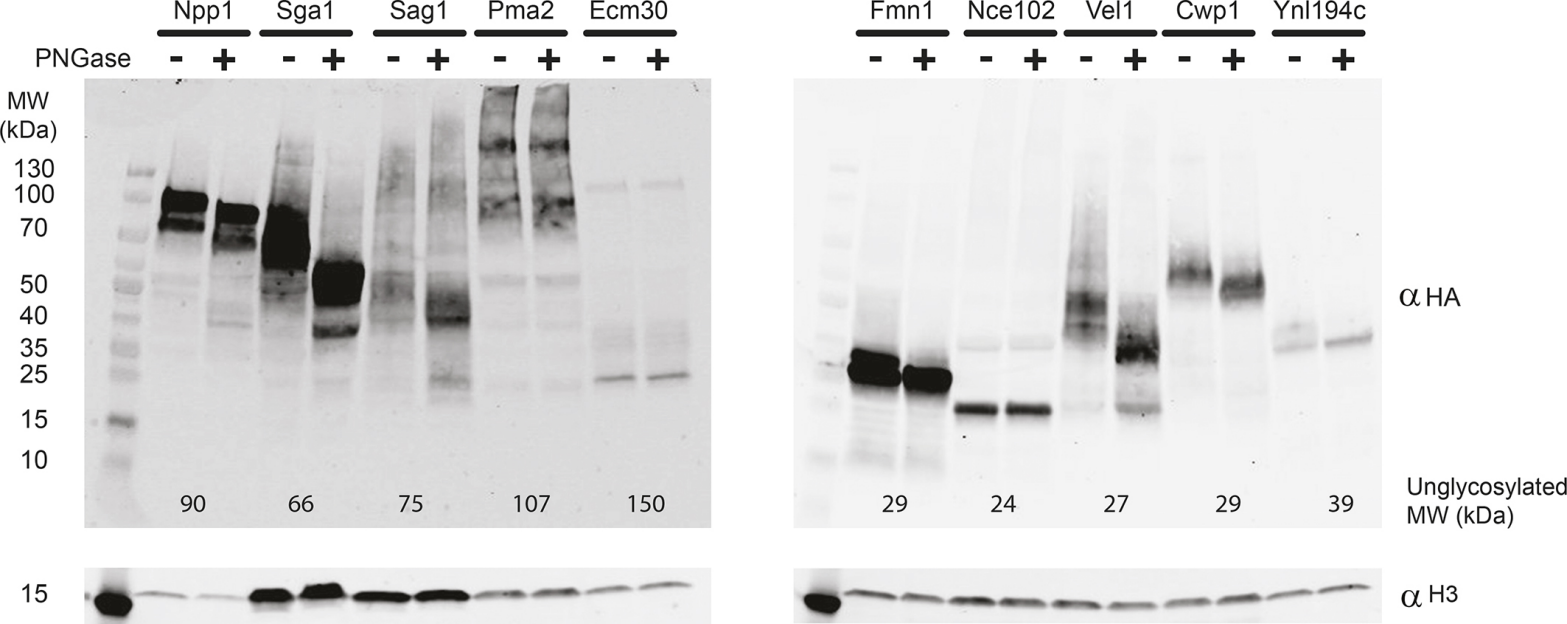
**Evaluation of the glycosylation state of HA-tagged proteins.** Whole-cell lysates from colonies of 10 known glycoproteins were treated with the pan-glycan removing enzyme, PNGase (+), or DDW (−) as control. Their molecular mass was subsequently resolved by western blot analysis using anti HA antibody. The calculated molecular mass of each protein, including the size of the 3xHA tag and L2 linker (4.9 kDa) is presented at the bottom of their respective lanes. Anti-histone H3 was used as a loading control. Blots shown are representative of two repeats.

To demonstrate the usability of the new library for western blot analysis, we chose to examine the glycosylation state of ten known glycosylated proteins using PNGase digestion for glycan removal. This analysis is based on the addition of the PNGase enzyme to the lysate resulting in the enzymatic digestion of the glycan tree and reduction of molecular mass of the proteins, which is easily visualized on an SDS-PAGE gel. The power of our collection is that instead of requiring an antibody for each detected protein, it is possible to assay multiple proteins at once using a single broadly used anti-HA antibody. To showcase the ease of use, we chose proteins of various molecular masses (Cao et al., 2014; Yeo et al., 2016; Zielinska et al., 2012) (Fig. 2). Six out of the 10 showed a clear molecular mass change after PNGase treatment (Npp1, Sga1, Fmn1, Vel1, Cwp1 and Ynl194c). Out of the four undetected proteins, three did not have a clear band, Pma2, Ecm30 and Sag1. Pma2 is a multi-pass membrane protein with nine or ten predicted transmembrane domains (Weill et al., 2019) and Ecm30 is a high-molecular-mass protein of 150 kDa, and therefore is difficult to resolve under the same conditions as the other proteins. Sag1 is an α-agglutinin, and therefore although it is expressed in the HA library under a constitutive promoter, it is likely to be unstable in the opposite mating type. Finally, Nce102 presented a similar band pattern with and without PNGase treatment at a molecular mass calculated for the unmodified protein. Hence, we might not have assayed this protein under conditions where it is in its glycosylated form.

Regardless, our assay demonstrates how the HA-tagged library facilitates rapid and systematic analysis of post-translational modifications across a diverse set of proteins, enabling insights into glycosylation and other modifications. In a broader sense, these results highlight how the availability of a full proteome library of HA-tagged proteins enables rapid size analysis for a selected set of proteins.

### Systematic quantification of protein abundance using N′ HA-tagged strains

To demonstrate the versatility of the N′-HA library in systematic assays to determine the amount of proteins in cells (which we denote as protein abundance, although it is not necessarily the native amount), we quantified protein levels. Given that all proteins are under the same constitutive *TEF1* promoter, which is one of the strongest promoters in yeast (Partow et al., 2010; Sun et al., 2012) and drives an expression that is stronger than all native promoters of tested proteins (Weill et al., 2018), our analysis identifies variations that are independent of promoter activity but rather influenced by factors such as RNA stability and protein degradation rates.

For this analysis, we chose a set of proteins selected for their varying expression levels in the *Nop1*-GFP library (Weill et al., 2018) despite being regulated by the same constitutive promoter.

**Fig. 3.**
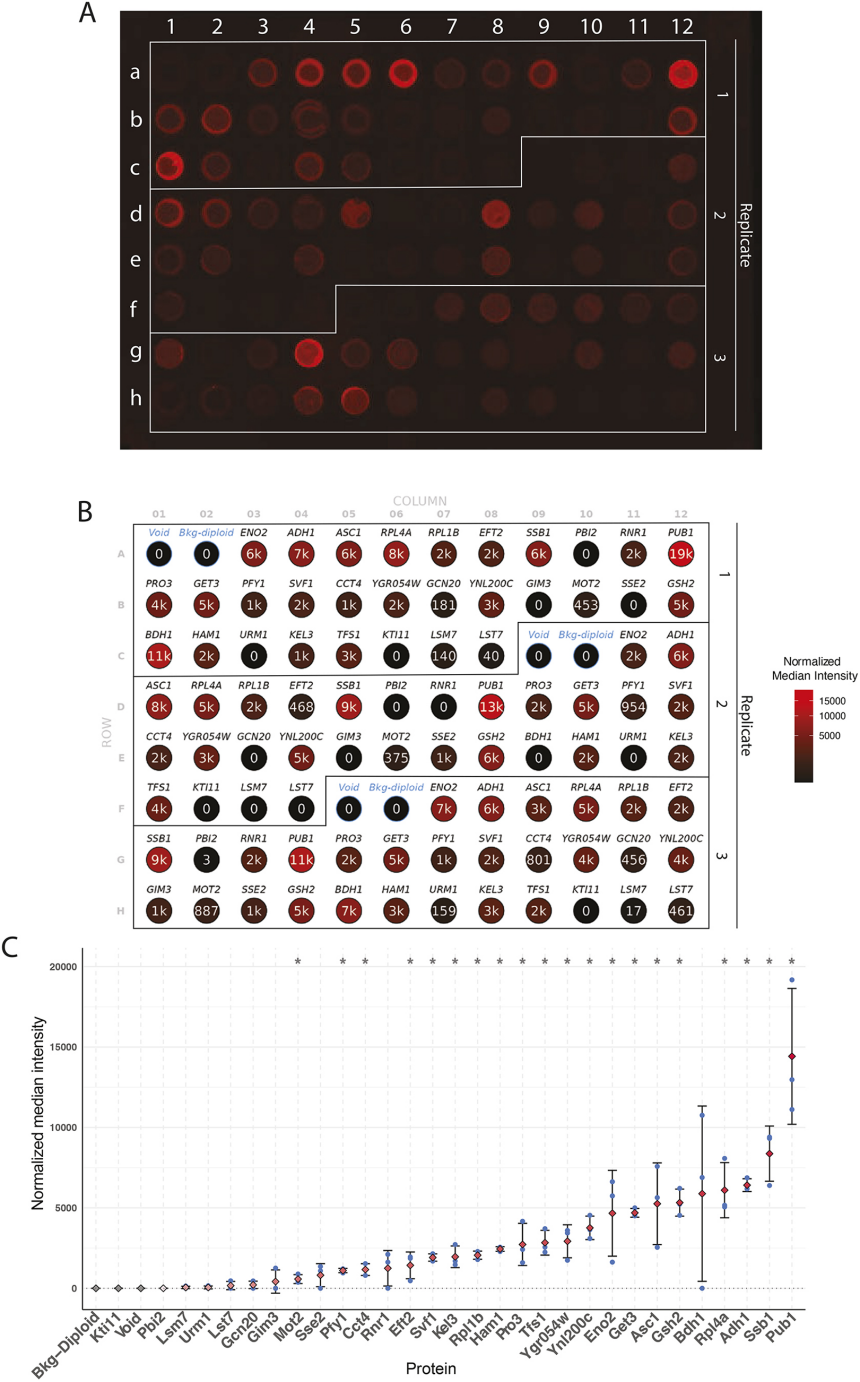
**Systematic quantification of protein abundance using the N′ HA strains.** (A) Image of a 96-well dot-blot with an anti-HA antibody, showing three replicates for each of 30 yeast strains and controls, one without cells and one using a strain not expressing any HA tagged protein. (B) Well annotation with median intensity values normalized to BCA protein concentrations. Circle color intensity represents the normalized median intensity for each well position. (C) A scatter plot displaying the average median intensity values over three replicates ranked in ascending order, with normalized median intensity for each strain shown as mean±s.d. as error bars. Asterisks represent *P*<0.05 (Wilcoxon rank-sum test).

To measure their abundance, we utilized high-throughput quantitative dot blotting (Fig. 3A,B). Calibrating the signal to the total amount of extracted proteins our analysis revealed clear differences in the abundance of the selected proteins, indicating that the N′ HA-tagged library enables effective detection of variations in protein levels (Fig. 3C). These results highlight the N′-HA library potential for reproducible and quantitative systematic assays for the analysis of cellular protein expression.

### Utilization of a genetically encoded affinity assay for visualizing protein localization

**Fig. 4.**
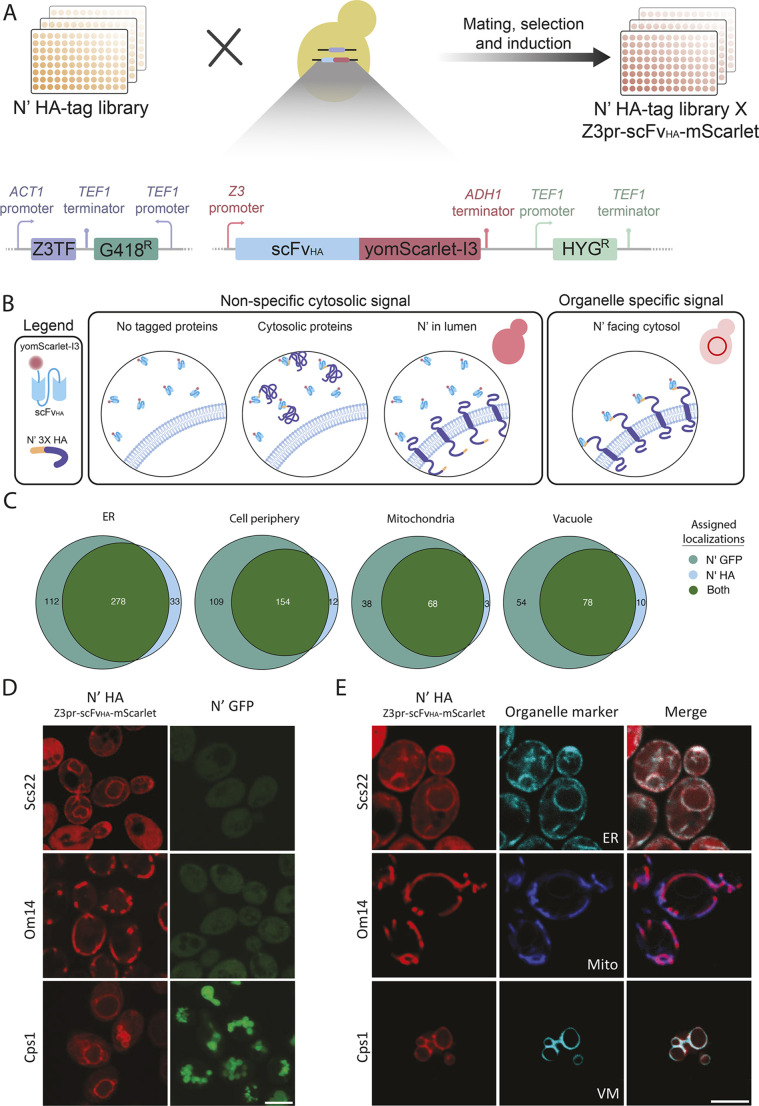
**Visualization of protein localization using a genetically encoded affinity assay.** (A) Schematic representation of the creation of the diploid library. The HA-tagged protein library was crossed against a strain expressing the Z3TF (transcription factor) under the ACT1 promoter and the scFv_HA_–yomScarlet-I3 (mScarlet) under the inducible Z3 promoter. As a result, a diploid library was created where each protein is N-terminally tagged with the 3xHA and constitutively expressed alongside the constitutive expression of the Z3TF. Upon exposure to β-estradiol, the Z3TF is activated, driving the expression of the scFv_HA_–mScarlet. The diploid library was subsequently imaged by confocal microscopy to detect localized mScarlet fluorescence signal. (B) Illustration of expected phenotypes for different N′ localization of HA-tagged proteins using the scFv_HA_–mScarlet, Z3TF system. Untagged proteins, cytosolic proteins and proteins with N′ facing lumens of organelle should show a cytosolic signal as the scFv_HA_–mScarlet is dispersed throughout the cytosol. Membrane proteins with cytosolic N′-HA will interact with the scFv_HA_–mScarlet and therefore present as a localized fluorescence signal of an organelle-specific nature. (C) Venn diagram of proteins exhibiting organelle specific signal in the *NOP1-*GFP and the HA×sc-Fv_HA_ libraries were analyzed for discrepancies in their localization. 65.7% of the GFP-tagged proteins that are localized to the ER also show a similar signal when tagged with HA. There was 56% correlation between GFP and HA for the cell periphery, 62.4% for mitochondria and 54.9% for the vacuole or vacuole membrane. Results are for two repeats. (D) Confocal microscopy images of representative strains that exhibited clear localizations to various organelles in the HA×sc-FV_HA_ library (red) despite not being clearly localized when tagged with GFP (green). Scs22 has a clear ER localization, Om14 a mitochondrial localization, and Cps1 vacuolar membrane localization. Diploid strains of both backgrounds were induced with β-estradiol and imaged after 3 h alongside their counterparts in the *NOP1-GFP* library. Scale bar: 5 µm. (E) Confocal microscopy images of the representative strains expressing organellar markers (cyan) showing full colocalization of the HA×sc-FV_HA_ strains (red) with the respective fluorescently labeled organelles. Sec63–mNeonGreen was used as a marker for the ER, MitoView405 was used as a mitochondrial marker and Vph1–mNeonGreen was used as a vacuole membrane (VM) marker. Scale bar: 5 µm. Images in D and E are representative of two repeats.

Although FP tag size and structure can influence protein structure and function, smaller tags usually do not enable *in vivo* visualization. To combine the advantages of a small tag with the capacity to visualize protein localization systematically, we utilized a system for monitoring the localization of the N′ HA-tagged proteins in live cells. To this end, we mated the HA library with a strain expressing a single-chain variable fragment that specifically binds the HA tag (scFv_HA_) fused to a fluorescent protein (Tsirkas et al., 2022; Zhao et al., 2019). The scFv_HA_ was fused to a yeast-codon optimized mScarlet-I3 fluorescent protein, which is a bright and rapidly maturing protein (Gadella et al., 2023). The scFv was expressed under the control of the inducible Z3 promoter on the background of its controlling Z3 transcription factor (Z3TF) (McIsaac et al., 2013; Ohira et al., 2017) in a BY4742 (Mat α) strain (Fig. 4A). Upon addition of β-estradiol to the medium, the Z3TF binds to the Z3 promoter, and induces the expression of scFv_HA_–mScarlet-I3 (hereafter dentoed scFv_HA_–Scarlet), thereby enabling the visualization of HA-tagged proteins. Having an inducible expression system allows the N′ HA proteins to fold, assemble and function while only having the small tag, but also be visualized, on demand, as the scFv is expressed.

Given that the scFV_HA_–Scarlet is expressed in the cytosol, it is not possible to use it to visualize cytosolic proteins nor proteins sequestered inside organelle lumens. However, membranal proteins whose N-terminus is facing the cytosol will bind the fluorophore to the surface of their respective organelles of residence, resulting in a localized fluorescent signal specific to the resident organelle (Fig. 4B).

To calibrate the optimal conditions for induction of the scFv_HA_–Scarlet, we measured its expression in the presence of different β-estradiol concentrations (0, 50, 100 and 1000 nM) at different times and chose 100 nM at a time range of 3–4 h for further analysis to allow Scarlet signal detection but prevent saturation of the signal (Fig. S2A,B). To validate these conditions, we manually picked library strains with N′-HA proteins from different cellular compartments that had a known topology of the N-terminus facing the cytosol [Alg7 (endoplasmic reticulum; ER), Gem1 (mitochondria), Pst2 (cell periphery), Tpd3 (cytosol) and Zrc1 (vacuole)], and mated them with the scFv_HA_–Scarlet strain. We then imaged these strains at 150 and 180 min after addition of 100 nM β-estradiol, validating their visualization at the expected organelle (Fig. S2C).

Using these optimized conditions, we then mated the entire HA library with the scFv_HA_–Scarlet strain and screened the diploid library for labeled proteins that exhibited organelle-specific signals. These proteins were compared to the *NOP1*-GFP library to identify any proteins whose localization might be better visualized by our system (Fig. 4C; Table S2). We focused on strains that displayed organelle-specific localization in our HA library and removed proteins with signal peptides (SPs) and mitochondrial-targeting sequences (MTSs) given that they would reside in the lumen or matrix of organelles clearly not compatible with our system. Our findings indicate that 65.7% of GFP-tagged proteins in the ER showed a similar signal when tagged with HA. Additionally, 56% of proteins in the cell periphery, 62.4% of mitochondrial proteins and 54.9% of vacuolar proteins had a similar localization with both tagging systems.

Interestingly, over 6% of the localizations observed in the HA library were novel, suggesting unique advantages of the HA library under specific conditions compared to the GFP library. This observation was manually validated by comparing strains showing new localizations in the HA library with those in the *NOP1*-GFP library. For example, Scs22, Om14 and Cps1 exhibited unique organelle-specific localization in the HA library. Scs22 is a well-characterized contact site protein, homologous to the mammalian VAP proteins, which is anchored to the ER membrane via a C′ transmembrane tail anchor (Manford et al., 2012). Om14 is a validated mitochondrial outer membrane protein (Burri et al., 2006) that could not be visualized using an N′ GFP tag but is now clearly visualized in mitochondria with the HA epitope (Fig. 4D,E). Cps1 is a vacuolar carboxy peptidase with an N′ transmembrane domain that is first targeted to the ER membrane and then transported to the vacuolar membrane. The C′ of Cps1 faces the lumen, where it is cleaved, releasing the catalytic subunit of the protein from the membrane to the vacuole lumen (Spormann et al., 1992). In the HA library, we found that the Scarlet signal of Cps1 is clearly marking the vacuolar membrane, whereas in the *NOP1*-GFP strain, the GFP signal localizes to the vacuolar lumen, indicating that the smaller HA tag allows for a more accurate depiction of the localization of Cps1 (Fig. 4D,E). These results underscore the benefits of using the HA tag as an additional exploratory system.

Building on the success of the scFv_HA_ system, we developed additional tools as an expansion of the scFv anti-HA toolkit. For colocalization studies, we generated yeast strains that can be crossed with the HA library, harboring either a vacuolar marker (Vph1–NG) or an ER marker (Sec61–NG) along with the scFv_HA_–yomScarlet-I3 system (Fig. S3A). In addition, we created two strains where the scFv_HA_ was fused to either the photoconvertible monomeric EOS3.1 (mEOS3.1) (Zhang et al., 2012) or the photo-switchable fast-forming Dronpa (ffDronpa) (Moeyaert et al., 2014) proteins (Fig. S3B,C). mEOS.1 is translated as a green fluorescence-emitting protein, and its emission can be converted into red fluorescence via illumination with a 405 nm laser. ffDronpa is also translated as a green fluorescence-emitting protein, and is bleached when excited, thus after imaging, existing ffDronpa molecules lose their fluorescence (switched off) whereas new ones are bright. Through illumination with a 405 laser, the switched-off ffDronpa are switched back on and can be imaged again. These advanced FPs, which change their fluorescence upon light exposure, allow for temporal and spatial tracking of protein dynamics and can also serve as control for the effect of the scFv_HA_ on protein turnover, significantly improving the versatility and depth of analysis possible with the library toolset.

## DISCUSSION

The optimal way to tag a protein is not uniform for all proteins. Depending on their structure, function, localization and other parameters, different proteins might be better suited to differing tagging strategies. Therefore, it is important to have a variety of libraries, using different tags representing the spectrum of sizes, charges and structures, to cover the entire proteome in a functional manner. In this study, we developed an N′-HA yeast proteome-wide library, generating the first epitope tag N-terminal library. The small and unstructured HA tag offers significant advantages by reducing the risk of interfering with protein folding, stability, assembly, localization or activity, for many proteins, while still enabling effective detection and functional assays. The utilization of the SWAT strategy allowed the creation of the N′ HA-tagged library, with high integration efficiency and proteome-wide coverage.

To demonstrate the versatility of the HA library, we showed its utility in the analysis of protein molecular mass and post-translational modifications rapidly and systematically. We also show the ability to quantify protein abundance enabling the discovery of factors that control protein expression beyond transcriptional regulation. Finally, the visualization of the N-HA protein by the scFv approach enables the *in vivo* characterization of the target proteins at its designated organelle.

Although the N′ HA-tagged protein bound to scFv_HA_-Scarlet system was effective for visualizing proteins in organelle membranes with cytosolic N-termini, it is not suitable for detecting proteins that are completely cytosolic or with the opposite topology where the N-terminus is inaccessible to the scFv_HA_. Two exceptions for this are proteins targeted to peroxisomes or the nucleus lumen, because their membrane import mechanisms can accommodate larger complexes (Meinecke et al., 2016; Rout and Aitchison, 2001). Indeed, by utilizing the library, we visualized luminal peroxisomal proteins, including Pex8, Pxp1 and Pcs60 and luminal nuclear proteins, such as Net1, Rsc6 and Nop10 in the correct cellular compartments.

Our reliance on a single constitutive promoter in the HA library does not account for natural promoter-specific expression variability, which could limit its application in studying dynamic gene regulation across different conditions. However, given that each library has its benefits and limitations, we foresee that this library will support a niche of assays that will benefit from this design. For other uses it is possible to use the SWAT approach to efficiently create a ‘seamless’ library where the HA tag is fused at the N′ but under the natural endogenous promoter and targeting signals (Yofe et al., 2016) or a C′ tagged library (Meurer et al., 2018).

Although the HA library can be used to study any protein of choice, it is probably most powerful for studying very small proteins where the large FP tag is a dramatic appendage. Recent insights into the role of small open reading frames (sORFs) provides additional dimensions to consider in proteomics research. sORFs, encoding proteins under 100 amino acids long, are often overlooked due to their size. However, they encode for small peptides that can have crucial biological functions, including roles in signaling, stress responses and translation regulation (Couso and Patraquim, 2017; Kastenmayer et al., 2006). Owing to their small size, it is crucial to minimize the size of appendages fused to them. Therefore, the N′-HA library provides an opportunity to systematically study these small peptides, potentially uncovering new functions or regulatory mechanisms. Upon analysis, 319 out of the 365 sORFs from the original SWAT library were retained in our final library, in line with the measured survival rate (Table S1).

Overall, our findings highlight the advantages of a proteome-wide N′ HA-tagged yeast library as a valuable tool for functional genomics. The applications demonstrated in this study, such as glycosylation detection, quantification of protein abundance and *in vivo* visualization of membrane proteins, illustrate the breadth of possibilities offered by this comprehensive library. Moving forward, the N′-HA library holds promise for further discoveries in yeast biology and might serve as a model for similar libraries in other organisms.

## MATERIALS AND METHODS

### Yeast strains and plasmids

All yeast strains used in this study are on the BY4741 laboratory strain (Backer Brachmann et al., 1998) and listed in Table S3. Strains were constructed using the lithium acetate-based transformation protocol (Gietz and Woods, 2002). All plasmids used are listed in Table S4, primers listed in Table S5 and antibodies listed in Table S6.

### Yeast library generation

SWAT library generation was performed as described previously (Weill et al., 2018). Briefly, a RoToR array pinning robot (Singer Instruments) was used to mate the parental N′ tag GFP SWAT library with the required donor strain (Table S3) and carry out the subsequent sporulation, and selection protocol to generate a haploid library selected for all the desired features. Growth of the library on YPGalactose (2% peptone, 1% yeast extract, 2% galactose) was used to induce SceI-mediated tag swapping, and subsequent growth on SD containing 5-fluoroorotic acid (5-FOA, Formedium) at 1 g/l, and required metabolic and antibiotic selections were used to select for strains that had successfully undergone the SWAT process. The resulting library resulted in each ORF N terminally tagged with a 3xHA tag (MYPYDVPDYAGYPYDVPDYAGSYPYDVPDYA) followed by the L2 linker (GGSSGGGGATENSS).

### Diploid strain generation

The N′ HA library was grown on SD supplemented with 200 μg/ml Nat (AB-102-25G; WERNER BioAgents) at 30°C overnight and the scFv_Anti HA_ -yomScarlet-I3, Z3TF donor strain (Table S3) was grown on YPD supplemented with 500 μg/ml geneticin (G418; G4185, Formedium) and 500 μg/ml hygromycin B (HYG; HYG5000, Formedium). Both were replicated onto a YPD plate and grown overnight at room temperature (RT). The mated strains were then replicated onto SD with monosodium glutamate (MSG) plates supplemented with Nat, G418 and HYG and grown overnight at 30°C. This step was repeated once more to select diploid strains containing the combination of desired traits.

### Protein extraction and SDS-PAGE for library accuracy validation

5 ml of cells with a 0.5 optical density at 600 nm (OD_600_) were collected by centrifugation at 3000 ***g*** for 3 min, washed with 1 ml of double-distilled water (DDW), resuspended in 200 μl lysis buffer containing 8 M urea, 50 mM Tris-HCl pH 7.5 and complete protease inhibitors (Merck), and lysed with a high-speed bead beater with glass beads (Scientific Industries) at 4°C for 10 min. 25 μl of 20% SDS was added before incubation at 45°C for 15 min. The bottom of the microcentrifuge tubes was then pierced, loaded into 5 ml tubes and centrifuged at 4000 ***g*** for 10 min to separate the lysate from the glass beads. The flow-through collected in the 5 ml tubes was transferred to a fresh 1.5 ml microcentrifuge tube and centrifuged at 20,000 ***g*** for 5 min. The supernatant was collected, and 4× SDS-free sample buffer (0.25 M Tris-HCl pH 6.8, 15% glycerol and 16% Orange G containing 100 mM DTT) was added to the lysates and incubated at 45°C for 15 min. Protein samples were then separated by SDS-PAGE using a 12% polyacrylamide gel and then transferred onto a 0.45 μm nitrocellulose membrane (Pall Corporation) using a Trans-Blot Turbo transfer system (Bio-Rad). Membranes were blocked in 3% bovine serum albumin (BSA) in phosphate-buffered saline (PBS) solution for 30 min at RT, incubated overnight at 4°C with rabbit anti-histone H3 (ab1791, 1:5000; Abcam) and mouse anti-HA (#901502 1:1000, BioLegend) diluted in a 2% (w/v) BSA in PBS solution containing 0.01% NaN_3_. After washing three times in TBST buffer (Tris-buffered saline with 0.1% Tween 20), membranes were then probed with secondary goat anti-rabbit-IgG antibody conjugated to IRDye680RD (#ab216777; Abcam) and 800CW goat anti-mouse-IgG (#ab216772; Abcam), both diluted 1:10,000 in 3% (w/v) milk in TBST solution for 1 h at RT. Blots were washed and imaged on the LI-COR Odyssey Infrared Scanner (ODY-2064). Raw blots can be found in Fig. S4.

### Library efficiency analysis

55 random colonies were assayed by PCR for the correct genomic integration of the HA cassette. Each colony was picked into 50 µl of NaOH 20 mM and boiled at 100°C for 25 min for DNA extraction. Extractions were centrifuged at 3200 ***g*** for 5 min and 2 µl were used for PCR analysis. PCR was done using GoTaq Green Master Mix (#M712B, Promega) and primers listed as in Table S5.

### Library coverage analysis

Images of agar plates of the N′ HA library were taken by a Canon digital camera. The images were subsequently processed by SGAtools v2.3 (Wagih et al., 2013) to measure the size and shape of each colony and establish a minimum size threshold for inclusion as a valid colony.

### Post-translational modification analysis

Yeast strains were grown overnight in YPD medium containing Nat at 30°C. The following day, the strains were back diluted to an OD_600_=0.2 and grown at 30°C for 4 h. Next, 2.5 OD_600_ of yeast cells were harvested, washed once in DDW, resuspended in 0.1 M NaOH and incubated for 5 min at RT. The cells were then centrifuged at 3000 ***g*** for 3 min. The pellets were subsequently assayed for glycosylation with the PNGase-F kit (New England Biolabs, P0704S) according to the manufacturer's protocol and resolved by SDS-PAGE. Raw blots can be found in Fig. S4.

### High-throughput quantitative dot-blot

#### Protein extraction

Yeast strain colonies were inoculated into a 1 ml polypropylene 96 deep-well plate, filled with 400 µl of YPD medium supplemented with 200 µg/ml of Nat as a selective agent. After inoculation, the plate was sealed with a breathable cover (AeraSeal BS-25, Excel scientific) and placed in a shaker incubator set to 30°C with shaking at 650 rpm for overnight growth. The following day the 96-well plate was spun down using a swing-out rotor (Eppendorf model 5810R) at 3000 ***g*** for 5 min. The resulting pellets (roughly 2 OD per sample) were washed once with TE buffer and stored at −20°C until further use. Subsequently, samples were resuspended in 220 µl of protein extraction buffer (8 M Urea, Tris-HCl pH 7.5). To break open the yeast cell wall, another 1 ml 96 deep-well plate was customized by drilling a 1 mm diameter hole at the bottom of each of the wells. Then, the punctured wells were carefully sealed with an aluminium sticker (PCR-AS-200, Axigen) before being filled with ∼100 µl of acid-washed glass beads (Merck cat. G8772) and 100 µl of Urea-suspended samples. Next, the wells were sealed with a second aluminum sticker, and the plate was mounted on a vortex with a flat adapter (Scientific Industries, Part No.504-0235-00) and vortexed for 15 min at maximum vibration frequency (3220 rpm) in a cold room. Then the bottom aluminum sticker was peeled off just before securing it atop a new 96-well, fully skirted PCR plate with adhesive tape. The tandem plates were spun at 800 ***g*** for 3 min using a swing-out centrifuge to transfer the extracted sample into the lower PCR plate. A new PCR plate was set up for the final preparation by adding 15 µl of 4× Laemmli buffer (supplemented with DTT) to each well. Then, 45 µl of the extracted samples were transferred to this plate, mixed by pipetting with the Laemmli buffer, sealed with a PCR sticker, and heated to 95°C for 10 min.

#### Dot blotting

For dot blotting, a nitrocellulose membrane, soaked in protein transfer buffer (Bio Prep, TB192), was placed on a sheet of Whatman blotting paper (no. 3) laid atop the bottom part of the Dot-blotter manifold. After soaking, the manifold's upper part was assembled and secured with clips. Then 35 µl of each sample was loaded into its designated position in the manifold using a multichannel pipette while the manifold was connected to a vacuum suction. The dotted membrane was then subjected to a Trans-Blot Turbo transfer system (Bio-Rad) to immobilize the protein samples, with a transfer program set to run for 7 min at 2.5A. Following immobilization, primary (anti-HA, Roche, 11867423001, 1:1000) and secondary (IRDye 800CW Goat anti-rat, Li-Cor, 92632219, 1:10,000) antibodies were applied according to standard western blotting protocols.

#### Image acquisition

The near-infrared fluorescence signals of the HA-tagged proteins were acquired from the dot-blot membrane using an Odyssey infrared imaging system (ODY-2064, Li-Cor Biosciences). The signal intensity was normalized through a BCA protein quantification assay (Thermo Fisher Scientific, 23225). Images were captured through Odyssey software (v3.0.21) with standardized scanning parameters, including a 169 µm resolution and intensity settings 5.0 for the 800 nm channel. The images were then exported in TIFF format to preserve the original pixel intensity values for subsequent analysis.

#### Signal enhancement

Prior to quantification, we systematically followed a quality control workflow to ensure robust and reproducible measurements. Dot-blot images were individually inspected for potential technical artifacts or poor signal quality that might compromise accurate quantification. Notwithstanding our high-quality imaging, we still implemented a two-step background correction in ImageJ/Fiji (v1.54f) to account for slightly uneven illumination: (1) a noise reduction using a Gaussian blur filter (σ=2 pixels) and (2) a local background estimation of the blurred image using the rolling-ball algorithm (radius=35 pixels) which was subsequently subtracted from the original image.

#### Signal quantification

Our custom Python software (ht-qdotblot) enables semi-automated quantification of signal intensity across the 96-well format. The software implements a grid-based quantification approach, where users initially register three reference points corresponding to the centers of the plate corners (wells A1, A12 and H1). The software then automatically overlays a virtual grid that can be fine-tuned through adjustments of well radius, spacing, and positioning. A grid-based approach allows for the selection of consistent region-of-interest across multiple images and experimental conditions. For each well, the software returns multiple intensity-based measurements, including median, mean, standard deviation mode, and minimum and maximum values. The data can be exported in CSV format for further analysis and visualization. Our GitHub repository contains the software source code and provides a guided tutorial for installation and usage on an example image (https://github.com/benjamin-elusers/HT-qDotblot).

#### Sample normalization and statistical analysis

To account for technical variations in protein loading and transfer efficiency, we employed a normalization strategy. Total protein content was quantified using the BCA assay (Thermo Fisher Scientific, cat. #23225), providing a normalization factor related to actual protein concentration in each well. Furthermore, we subtracted the background values of two internal controls (buffer-only and untagged yeast) from the normalized median intensity signals. The final sample integrated intensities were calculated as the mean of background-subtracted normalized median intensities across replicates, which were scattered on the plate to avoid any spatial bias in the quantification. For the 32 samples, we report the individual integrated intensities along with their mean±s.e.m. from three independent replicates. Statistical significance was calculated by the Wilcoxon rank-sum test.

### Confocal microscopy

For high-throughput screening of the full library, cells were moved from agar plates into liquid 384-well plates using the RoToR bench-top colony arrayer (Singer Instruments). Liquid cultures were grown overnight in synthetic medium with 2% glucose (SD) in a shaking incubator (LiCONiC Instruments) at 30°C. A Tecan freedom EVO liquid handler (Tecan), which is connected to the incubator, was used to back-dilute the strains to ∼0.25 OD_600_ in plates containing SD with 100 nM β-estradiol. Plates were then transferred back to the incubator and were allowed to grow for 4 h at 30°C to reach logarithmic growth phase. The liquid handler was then used to transfer strains into glass-bottom 384-well microscope plates (Azenta Life Sciences) coated with 0.25 mg/ml concanavalin A (Sigma-Aldrich) to allow cell adhesion. Wells were washed twice in SD to remove floating cells and reach a cell monolayer. Plates were then automatically moved by a KX-2 robotic arm (Peak Robotics) into an automated inverted spinning disk microscope system (Olympus) and imaged using a 60× air lens (UPlanFLN, NA 0.9).

For all other microscopy-based figures, images were obtained using an automated inverted fluorescence microscope system (Olympus) containing a spinning disk high-resolution module (Yokogawa CSU-W1 SoRa confocal scanner with double micro lenses and 50 μm pinholes). Several planes were recorded using a 60× oil lens (NA 1.42) and with a Hamamatsu ORCA-Flash 4.0 camera. Fluorophores were excited by a laser and images were recorded in three channels: GFP (excitation wavelength 488 nm, emission filter 525/50 nm), mCherry/mScarlet/FM™4-64 (excitation wavelength 561 nm, emission filter 617/73 nm) and MitoView™405 (excitation wavelength 405 nm, emission filter 447/60). Image acquisition was performed using scanR Olympus soft imaging solutions version 3.2. and fluorescence quantification was performed using scanR analysis 3.2. Images were transferred to ImageJ, for slight contrast and brightness adjustments to each individual panel. Images were manually inspected using Fiji-ImageJ software (Schindelin et al., 2012).

## Supplementary Material

10.1242/joces.263848_sup1 Supplementary information

Table S1. Surviving colonies from SWAT procedure present in the final HA library

Table S2. Assigned localizations for HA tagged proteins

Table S3. A list of yeast strains used in this study.

Table S4. A list of plasmids used in this study.

Table S5. A list of primers used in this study.

Table S6. A list of antibodies used in this study.

Table S7. Efficiency check - PCR tests.
