# Supplementary material for: Creation and validation of a proteome-wide yeast library for protein detection and analysis
Source: J Cell Sci. 2025 Sep 1;138(16):jcs263848. doi: 10.1242/jcs.263848 (PMC12450460; doi:10.1242/jcs.263848)
Supplement: Supplementary information [file joces-138-263848-s1.pdf]

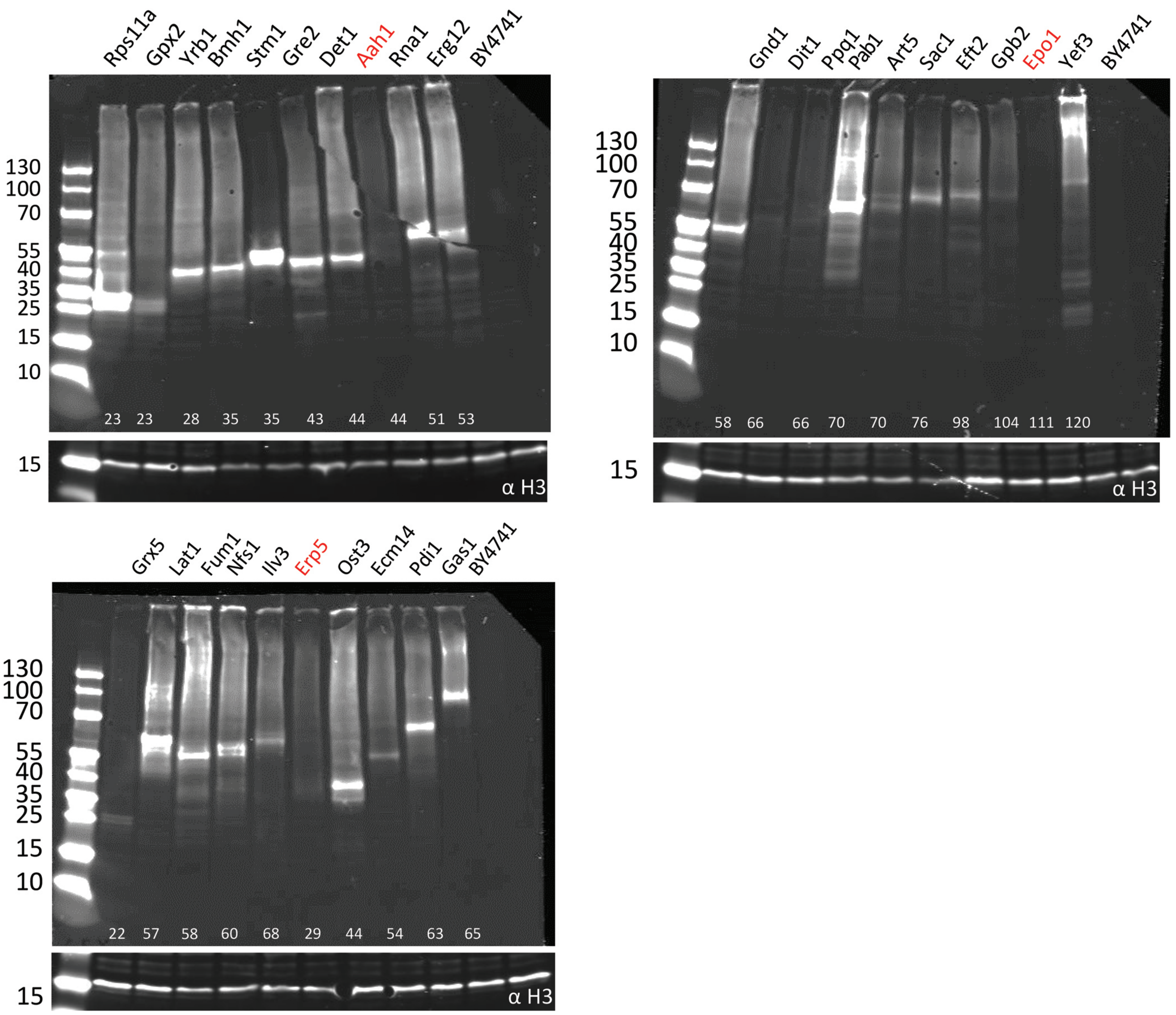

Fig. S1. Verification of the N' HA-tagged yeast library using Western blot analysis.
Western blots of selected HA-tagged yeast strains to verify successful, in frame, integration of the HA tag. Each lane represents a different HA-tagged protein from the library, with anti-HA antibodies used to detect the presence and correct size of the tagged proteins. Control lanes include a non-HA-tagged yeast strain as a negative control (BY4741) to confirm specificity of the anti-HA antibody. Molecular weight (MW) markers indicate size ranges for each HA-tagged protein. The calculated Mw of each protein, including the size of the 3xHA tag and L2 linker (4.9 KDa) is presented at the bottom of their respective lanes. Bands at expected sizes confirm that most proteins are correctly tagged and expressed. Anti histone H3 was used as a loading control. Protein names in red indicate lack of a clear band.

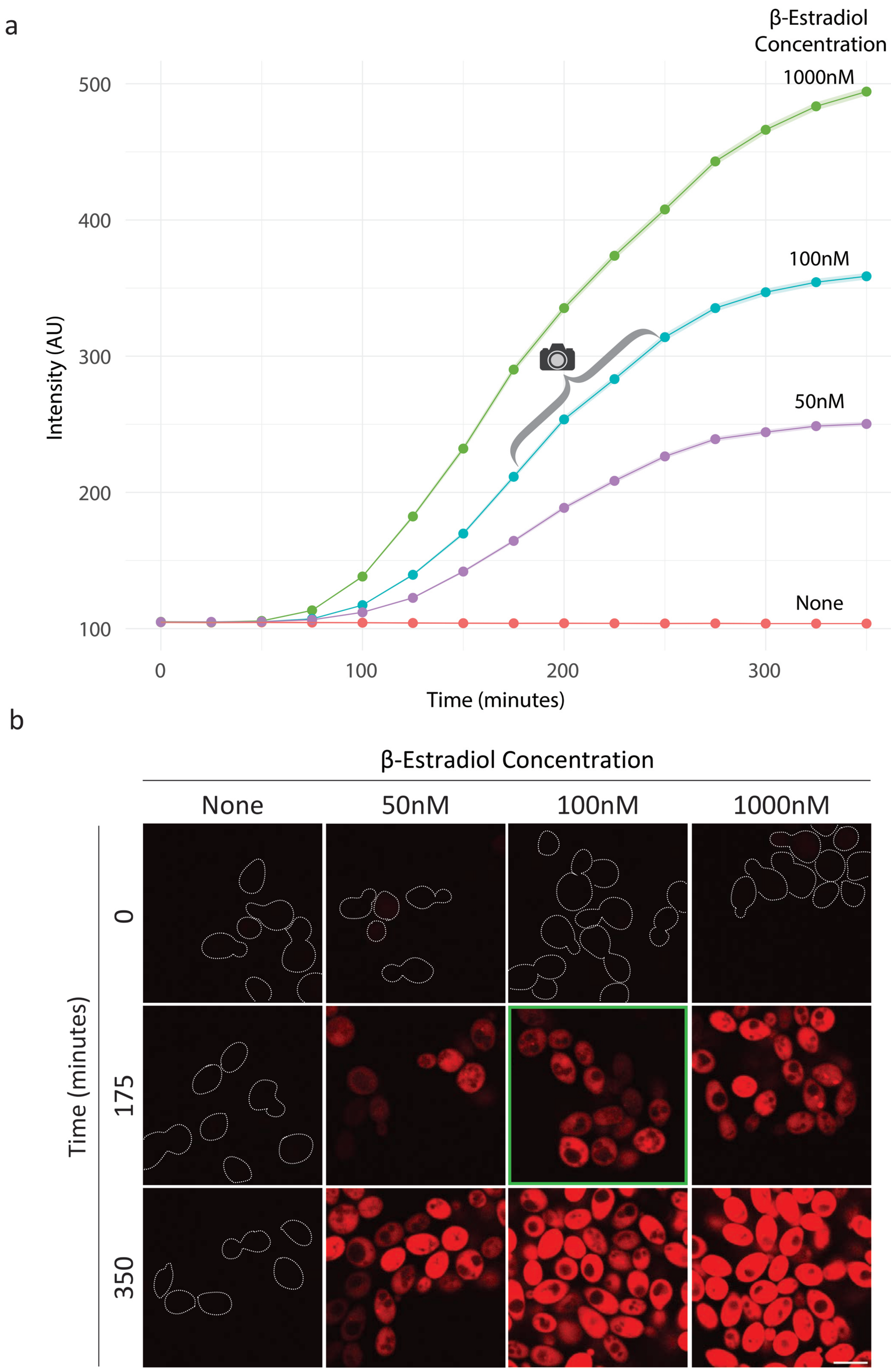
a
β-Estradiol
Concentration
1000nM
100nM
50nM
None
500
400
300
200
100
Intensity (AU)
0
100
200
300
Time (minutes)
b
β-Estradiol Concentration
None
50nM
100nM
1000nM
Time (minutes)
0
175
350

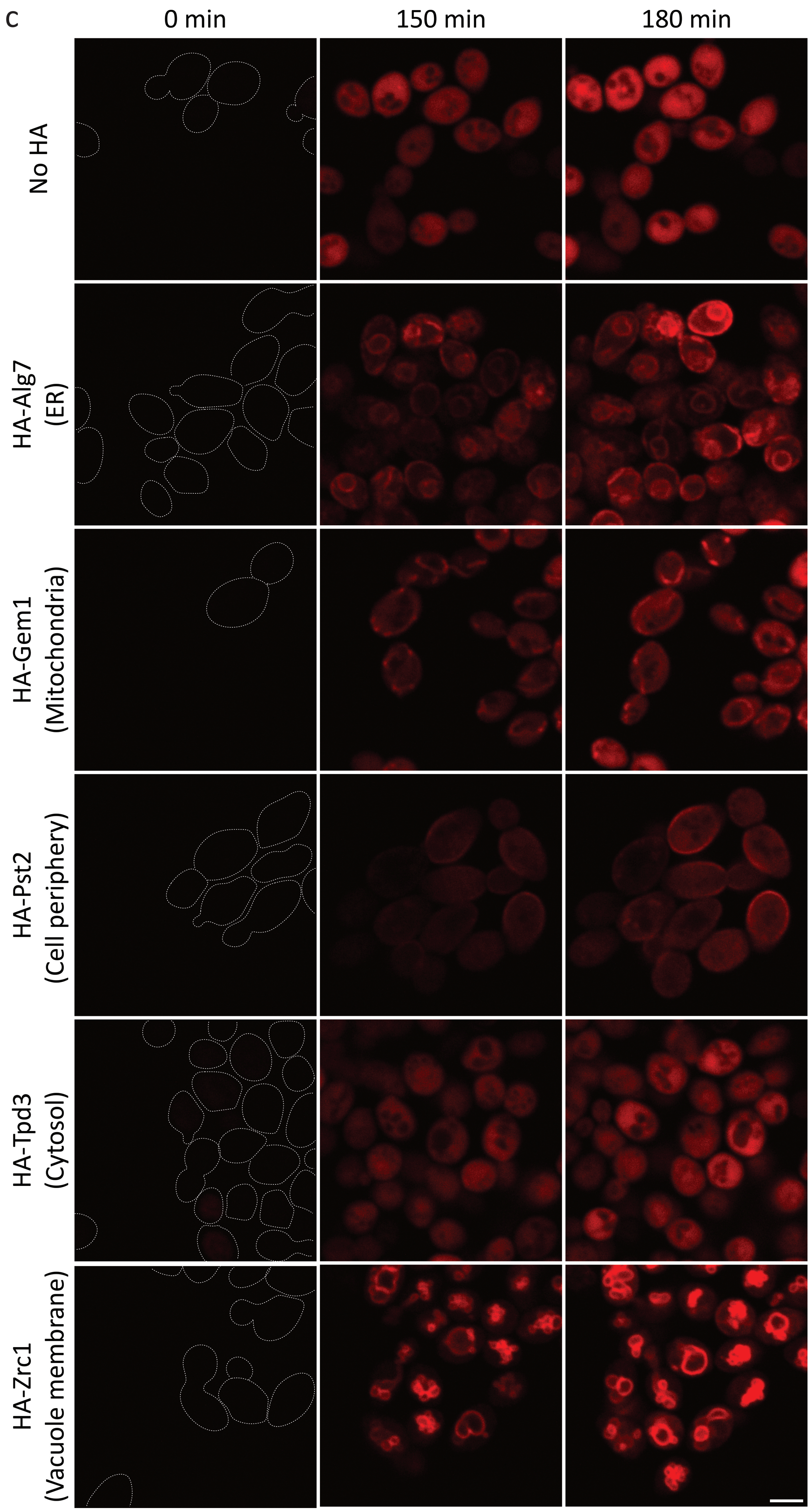

Fig. S2. Calibration and optimization of the scFv anti-HA system for imaging.

(a) Plot of intensity (arbitrary units, AU) over time (minutes) in yeast strains containing the $scFv_{HA}$-Scarlet system induced with β-estradiol at concentrations of 50nM, 100nM, and 1000nM. A strain without β-estradiol was used as a control (none). The plot highlights the optimal time and β-estradiol concentration (indicated with brackets and a camera icon) for achieving a coherent visualized signal (100nM, ~180-250min).

(b) Representative confocal images of yeast strains with the $scFv_{HA}$-mScarlet system at various time points and β-estradiol concentrations from (a). Images demonstrate increasing fluorescence intensity with higher concentrations and longer induction times. Scale bar = 5 µm.

(c) Representative confocal images of diploid yeast strains (N'-HA + $scFv_{HA}$-mScarlet) with HA-tagged proteins localized to various organelle membranes: Alg7 to the endoplasmic reticulum (ER), Gem1 to mitochondria, Pst2 to the cell periphery, Tpd3 in the cytosol, and Zrc1 to the vacuole membrane. Images were taken at 0, 150, and 180 minutes after β-estradiol induction to monitor changes in signal intensity. The fluorescent signals confirm the accurate localization of HA-tagged proteins across these compartments. Scale bar = 5 µm.

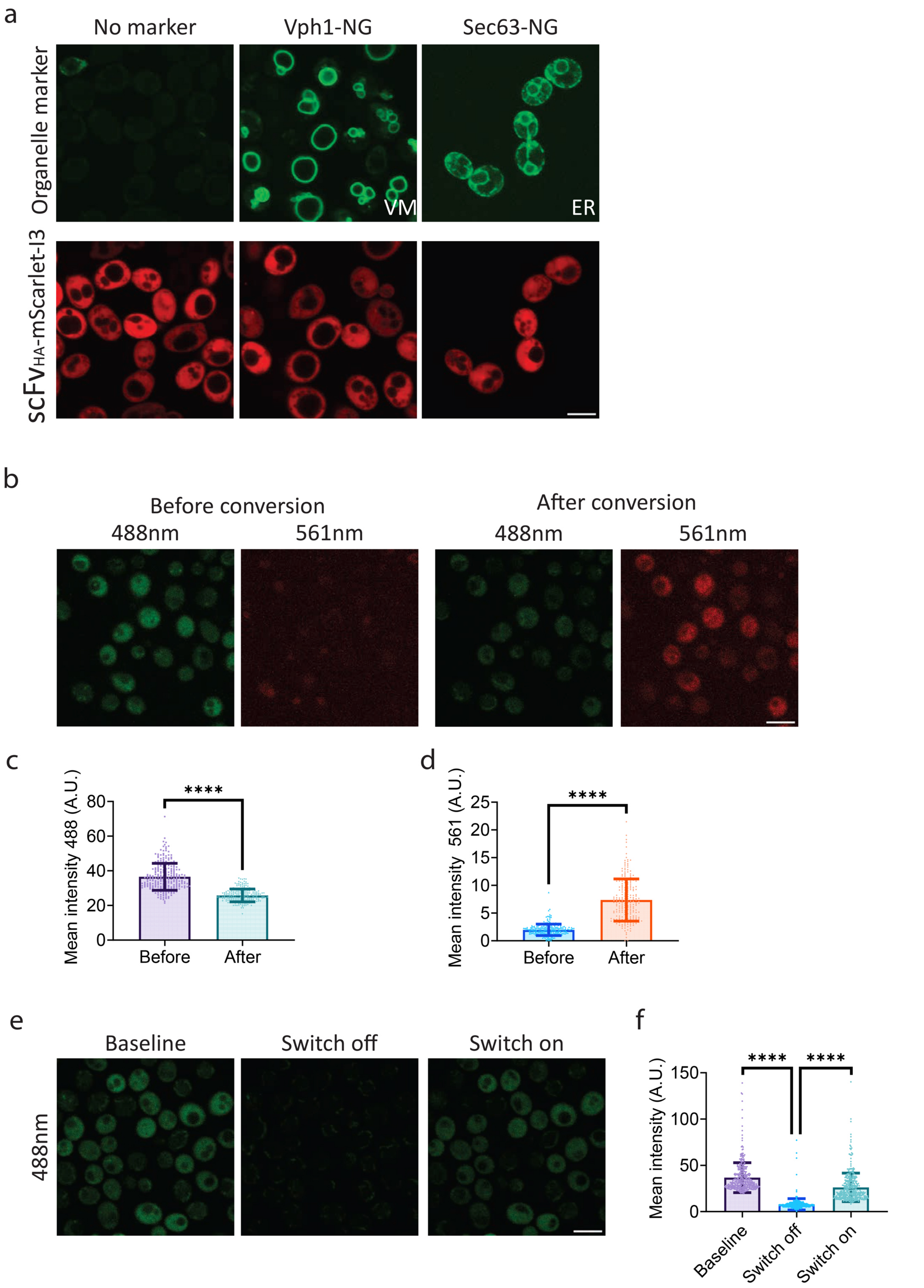

Fig. S3. Expansion of the scFv anti-HA toolkit with organellar markers, photoconvertible and photo-switchable proteins.
(a) Confocal images of diploid yeast strains containing the $scFv_{HA}$-mScarlet system (red) alongside genome-integrated organelle markers (green), as used in figure 4e: Vph1 (vacuole membrane, VM) and Sec63 (endoplasmic reticulum, ER). Scale bar = 5 µm.
(b) Confocal images of a yeast strains containing $scFv_{HA}$-mEOS3.1, a photoconvertible fluorophore, excited by 488 nm and 561 nm lasers. Photoconversion from green to red, after illumination by a 405nm laser, demonstrating the expansion of the toolkit's versatility for dynamic protein tracking. Scale bar = 5 µm.
(c) Quantification of mean intensity of cells excited at 488 nm from (b). n=2 fields. 256 and 225 individual cells were quantified before and after conversion, respectively, and analyzed by unpaired t-test. pv<0.0001. Bars = mean ± SD.
(d) Quantification of mean intensity of cells excited at 561 nm from (b). n=2 fields. 256 and 225 individual cells were quantified before and after conversion, respectively, and analyzed by unpaired t-test. pv<0.0001. Bars = mean ± SD.
(e) Confocal images of a yeast strain containing $scFv_{HA}$-ffDronpa, a photo-switchable fluorophore, imaged at 488 nm for the first time (baseline), after bleaching (switch off), and after subsequent excitation at 405 nm (switch off). These images demonstrate that $scFv_{HA}$-ffDronpa can be efficiently toggled between fluorescent states, allowing controlled imaging in live cells. Scale bar = 5 µm.
(f) Quantification of mean intensity of cells excited at 488 nm from (e). n=2 fields. 376 cells were quantified and analyzed by paired t-test. pv<0.0001. Bars = mean ± SD.

a

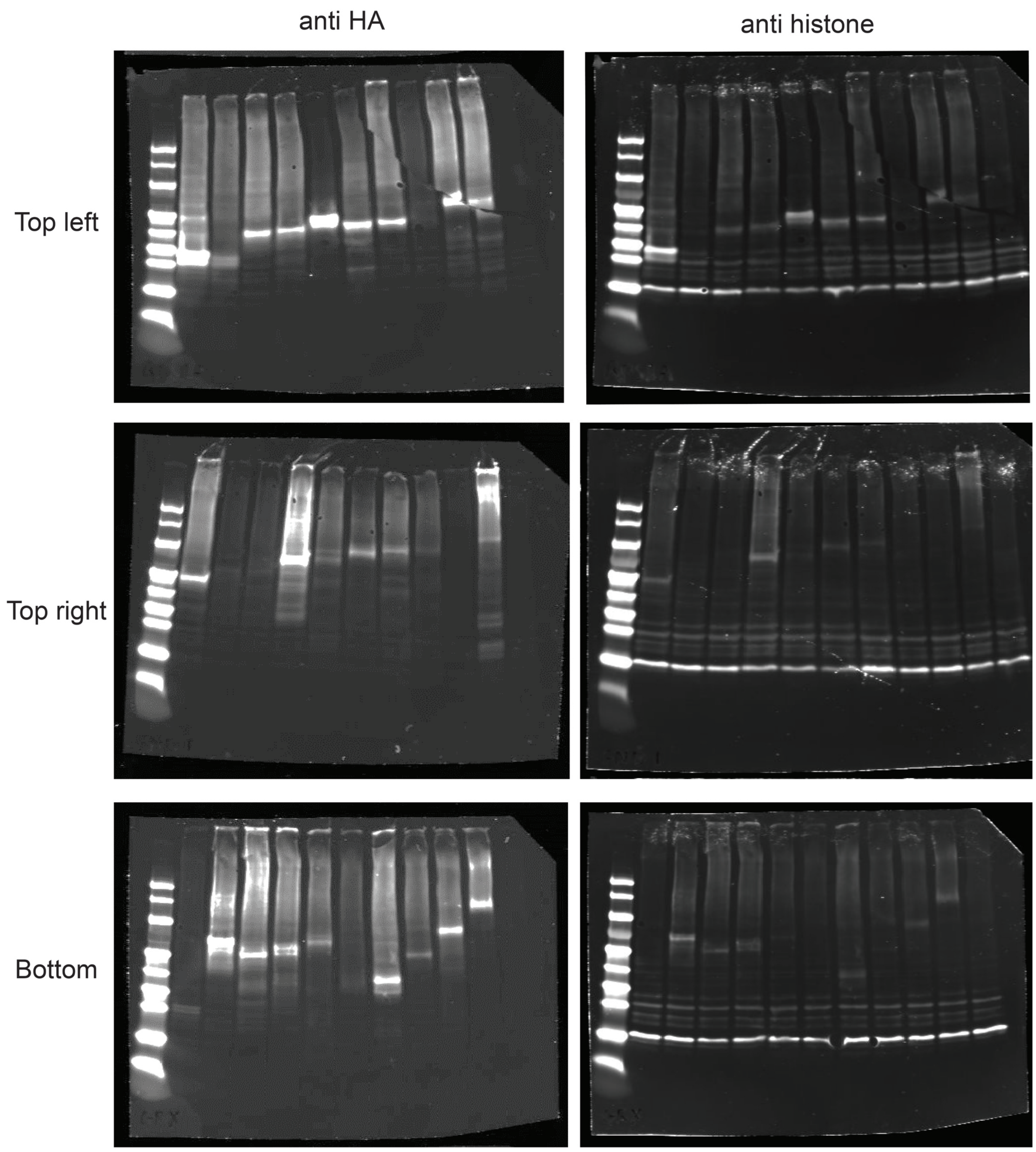

b

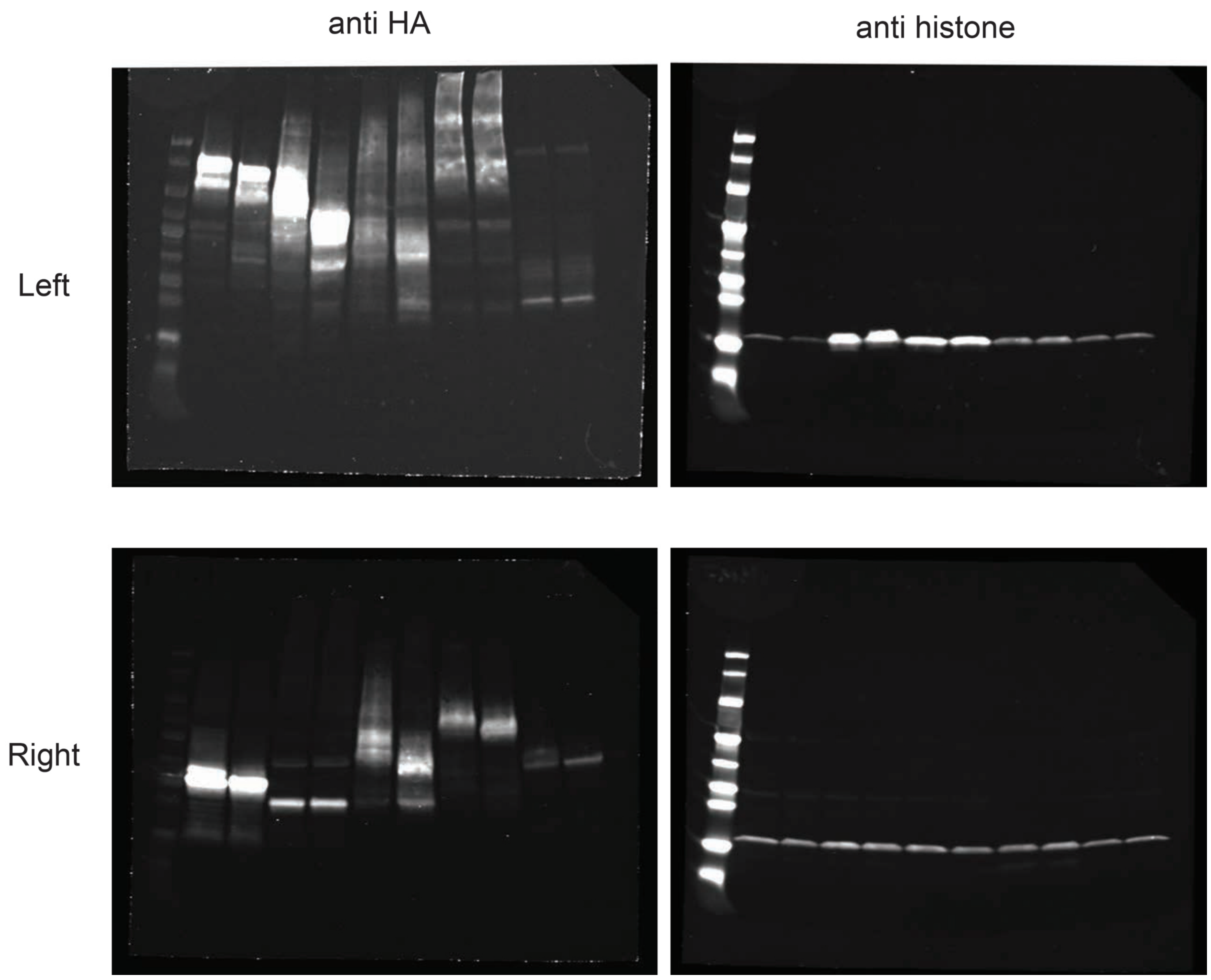

Fig. S4. Western blot transparency.
Original western blot data. **(a)** Blots of Fig. S1. Text on the left indicates their position in the correlating figure.**(b)** Blots of figure 2. Text on the left indicates their position in the correlating figure.

Journal of Cell Science • Supplementary information

Table S1. Surviving colonies from SWAT procedure present in the final HA library

Available for download at
https://journals.biologists.com/jcs/article-lookup/doi/10.1242/jcs.263848#supplementary-data

Table S2. Assigned localizations for HA tagged proteins

Available for download at
https://journals.biologists.com/jcs/article-lookup/doi/10.1242/jcs.263848#supplementary-data

Table S3. A list of yeast strains used in this study.

Available for download at
https://journals.biologists.com/jcs/article-lookup/doi/10.1242/jcs.263848#supplementary-data

Table S4. A list of plasmids used in this study.

Available for download at
https://journals.biologists.com/jcs/article-lookup/doi/10.1242/jcs.263848#supplementary-data

Table S5. A list of primers used in this study.

Available for download at
https://journals.biologists.com/jcs/article-lookup/doi/10.1242/jcs.263848#supplementary-data

Table S6. A list of antibodies used in this study.

Available for download at
https://journals.biologists.com/jcs/article-lookup/doi/10.1242/jcs.263848#supplementary-data

Table S7. Efficiency check - PCR tests.

Available for download at
https://journals.biologists.com/jcs/article-lookup/doi/10.1242/jcs.263848#supplementary-data

Journal of Cell Science • Supplementary information
